# Successful Applications of NIR Spectroscopy and NIR Imaging in the Food Processing Chain

**DOI:** 10.3390/foods12163041

**Published:** 2023-08-13

**Authors:** Giacomo Squeo, José Manuel Amigo

**Affiliations:** 1 Department of Soil Plant and Food Sciences, University of Bari Aldo Moro, Via Amendola 165/A, 70126 Bari, Italy; 2 Department of Analytical Chemistry, University of the Basque Country UPV/EHU, P.O. Box 644, 48080 Bilbao, Spain; josemanuel.amigo@ehu.eus; 3 IKERBASQUE, Basque Foundation for Science, 48011 Bilbao, Spain

Forty years ago, Near InfraRed (NIR) was considered a sleeping technique among the spectroscopic ones. Thanks to the technological advances suffered in recent decades, we can say that NIR is now a consolidated technique, and with rapidly increasing applications. After attracting more attention inside the laboratories, NIR Spectroscopy (NIRS) moved out of them, being used in companies (in-line and on-line process control), various fields (airborne devices and handheld devices), and even space (satellites). Many factors have contributed to this success story, whose end is, we believe, still far from being written. Several of the most important are cited here: the economic accessibility of powerful devices, the technical evolution of the instrumentation, and the acceptance of the use of chemometrics, whose impact in the field on NIRS is absolutely needed due to the chemical and physical features of NIR radiation. Other factors, such as the miniaturization of the instruments and the association with imaging techniques, which occurred with the launch of the 4th industrial revolution and the development of the Industry 4.0 paradigm, have been pivotal.

The present Special Issue was developed within this framework with the aim of collecting scientific articles showing the potential of NIRS, coupled or not with imaging techniques, for new, diverse, and innovative real industrial applications. The impressive flexibility of NIRS is shown in the collected articles, which span from the in-line estimation of fat marbling in whole beef striploin by NIR hyperspectral imaging [1] to the in-line application of NIRS for quality monitoring in a large-scale cheese production plant [2]; from the discrimination of normal vs. dark, firm, and dry (DFD) beef meat and the prediction of quality traits [3] to the amino acid profiling of quality protein maize [4]; from the tracking of sugar content distribution of white strawberry by NIR hyperspectral imaging [5] to the estimation of black root mould infection in apples [6], and the detection of the “Dangshan” physiological disease of pears [7].

As the readers will appreciate, interesting applications on both animal and vegetal products are reported, and in-line or post-harvest scenarios are considered. Consequently, various multivariate data analysis approaches were used to treat the different data. In conclusion, we are confident that the contributions collected in the Special Issue “Successful Applications of NIR Spectroscopy and NIR Imaging in the Food Processing Chain” will provide interesting insights to those interested in using and developing analytical and process solutions based on NIRS.
